# Shenfu Injection for Intradialytic Hypotension: A Systematic Review and Meta-Analysis

**DOI:** 10.1155/2014/279853

**Published:** 2014-12-22

**Authors:** Yenan Mo, Xusheng Liu, Xindong Qin, Jing Huang, Zhiren He, Junjie Lin, Qinqing Hu, Youqing Cai, Zhuangzhu Liu, Lixin Wang

**Affiliations:** ^1^The Second Clinical Medical School, Guangzhou University of Chinese Medicine, Guangzhou 510405, China; ^2^Nephropathy Department, The Second Clinical College of Guangzhou University of Chinese Medicine, Guangzhou Provincial Hospital of Chinese Medicine, Guangzhou 510120, China; ^3^Emergency Department, The Second Clinical College of Guangzhou University of Chinese Medicine, Guangzhou Provincial Hospital of Chinese Medicine, Guangzhou 510120, China

## Abstract

*Objective*. To evaluate the effectiveness and safety of Shenfu injection (SFI) for intradialytic hypotension (IDH).* Methods*. A systematic review of data sources published as of April 2014 was conducted. These included the Cochrane Central Register of Controlled Trials (2014 issue 4), Pubmed, Embase, CBM, CNKI, VIP, and Wangfang Data. Randomized controlled trials (RCTs) involving SFI for treatment and prevention of IDH were identified. Two researchers independently selected articles, extracted data, assessed quality, and cross checked the results. Revman 5.2 was used to analyze the results.* Results*. Eight RCTs were included. The meta-analysis indicated that compared with conventional therapies alone, SFI could elevate systolic blood pressure (SBP), increase the clinical effective rate, decrease the incidence of hypotension, increase serum albumin (ALB) levels, and reduce C-reactive protein (CRP) levels without serious adverse effects.* GRADE Quality of Evidence*. the quality of SBP, the effective rate, ALB, and CRP were low, and hypotension incidence and DBP were very low.* Conclusions*. SFI is more effective than conventional therapies for prevention and treatment of IDH. However, a clinical recommendation is not warranted due to the small number of studies included and low methodology quality. Multi-center and high-quality RCTs with large sample sizes are needed to provide stronger evidence.

## 1. Introduction

Intradialytic hypotension (IDH), the most frequent complication of hemodialysis (HD) treatment, occurs in ~20–30% of all dialysis sessions [1]. IDH is defined as a reduction in systolic blood pressure (SBP) by ≥20 mmHg or a reduction in mean arterial pressure (MAP) by ≥10 mmHg associated with symptoms such as dizziness, blurred vision, cramps, and fatigue [2].

The etiology of IDH is multifactorial. IDH occurs as a consequence of a mismatch between ultrafiltration and plasma refilling rates, coupled with a decline in cardiac function, changes in serum electrolyte concentrations, autonomic dysfunction, imbalance of vasoactive agents, dialysate temperature, and an immune response to dialysis [3]. IDH is independently associated with higher mortality in hemodialysis patients, contributes to a variety of clinical events such as stroke [4], mesenteric ischemia [5], cardiac infarction, and arrhythmias [6], and impairs the ability to reach the required dry weight, which will lead to fluid overload, hypertension, and left ventricular hypertrophy [7]. Moreover, IDH can accelerate decline in residual renal function [8] and cause occlusion of arteriovenous fistula [9].

Several strategies can be used to mitigate the frequency of IDH, with variable levels of success. These include general measures such as limiting interdialytic weight gain, increasing dry-weight, and avoiding food intake during dialysis [10], as well as sodium and ultrafiltration profiling [11], avoidance of hypocalcaemia [12], blood volume and blood temperature monitoring [13], biofeedback [14], midodrine [15] and carnitine [16], convective renal replacement therapies [17], and longer and more frequent dialysis [18, 19]. However, development of novel, safe, and effective treatments is still necessary; traditional Chinese medicine (TCM) may offer new insight.

In TCM, the pathogenesis of IDH is the collapse of qi resulting from massive loss of blood, leading to exhaustion of yin and collapse of yang. The therapy follows the important principle that visible blood cannot be generated promptly, while intangible qi should be rescued immediately [20]. Shenfu injection originated from Shenfu decoction, a well-known traditional Chinese herbal prescription recorded in Jishengfang (Yan's Prescriptions for Rescuing Lives) by Yan Yonghe in the 1250s [21]. Shenfu decoction consists of two Chinese herbal medicines,* Radix Ginseng *(ginseng) and* Radix Aconiti Lateralis Preparata* (prepared aconite root). The former can greatly tonify the original qi to engender blood, while the latter can restore yang and prevent collapse. The main active components of SFI are extracts of ginsenosides and higenamine [22]. Higenamine can stimulate *β*-adrenergic receptors in a manner similar to isoproterenol to directly increase SBP and myocardial beat frequency and amplitude to enhance myocardial contractility and improve atrioventricular conduction by strengthening the excitability of the sinoatrial node and atrioventricular node [23]. Ginsenosides can promote synthesis and release of prostacyclin, dilate coronary and reduce peripheral vascular resistance, and relieve myocardial ischemia [24]. SFI attenuates calcium influx, accelerates the clearance of oxygen radical, and inhibits the production of lipid peroxide, in order to antagonize ischemic reperfusion injury [25]. Clinical trials showed that SFI exerted a bidirectional effect on blood pressure, but the mechanism remains unknown [26, 27]. Therefore, SFI is used widely in treating shock [28], myocardial infarction [29], arrhythmia [30], and heart failure [31].

SFI has been used alone or in combination with routine therapies for treatment of IDH in China based on its effectiveness in observational studies and randomized controlled trials. However, the evidence of the effectiveness and safety of SFI has not been assessed systematically. The objective of this review is to evaluate the clinical effectiveness and safety of SFI for the treatment and prevention of IDH.

## 2. Methods

### 2.1. Inclusion Criteria

#### 2.1.1. Types of Study

All randomized controlled clinical trials (RCTs) that assessed the effect of SFI as adjunct therapy for IDH patients were included, regardless of publication status and language. We did not include quasi-RCTs, unequal RCTs, cluster RCTs, single RCTs, or cross-over-design RCTs.

#### 2.1.2. Types of Participant

The types of participant included long-term regular HD patients who had experienced repeated episodes of IDH without age or gender restriction. The diagnostic criteria were established as (1) K/DOQI guideline: a decrease in SBP ≥20 mmHg or a decrease in MAP ≥10 mmHg associated with dialysis-related hypotension symptoms [2]; (2) blood purification, 3rd edition guideline: SBP <90 mmHg or a decrease in SBP ≥20 mmHg from prehemodialysis [40]. No patient received antihypertensive drugs or any other intervention that could influence blood pressure before dialysis.

#### 2.1.3. Types of Intervention

The treatment groups included: SFI, used alone or in combination with conventional therapies, regardless of the dose, time-point, or duration of administration. The control group included conventional therapies, such as reducing the blood flow or ultrafiltration rate and use of vasoactive drugs.

#### 2.1.4. Outcomes

*(1) Primary Outcomes*. SBP, diastolic blood pressure (DBP), adverse events.

*(2) Secondary Outcomes*. The clinical effective rate, hypotension incidence and serum albumin (ALB), and C-reactive protein (CRP) levels. Clinical effectiveness was defined as follows: (1) markedly effective: SBP increased by ≥20 mmHg or SBP by >90 mmHg or MAP increased by ≥20 mmHg compared with pretreatment, with no hypotension-related symptoms, and dialysis was completed smoothly; (2) effective: SBP increased by 10–20 mmHg or SBP by >90 mmHg or MBP increased by 10–20 mmHg compared with pretreatment, with no obvious symptoms of low blood pressure, and dialysis to be completed by adjusting the dialysis program; (2) ineffective: blood pressure did not rise or continued to decline, SBP dropped to <90 mmHg, with significant symptoms of low blood pressure requiring vasopressors, volume expansion, and other drug treatment to maintain blood pressure, or interruption of dialysis.

### 2.2. Search Strategy

#### 2.2.1. Electronic Search

Cochrane Central Register of Controlled Trials, Pubmed, Embase, CBM, CNKI, VIP, and Wangfang Data were searched up to April 2014. The following search terms were used as subject headings and keywords: dialysis, hemodialysis, hematodialysis, haemodialysis, shenfu, shen-fu, hypotension, IDH, low blood pressure, and blood pressure variability.

#### 2.2.2. Manual Search

Proceedings of the Blood Purification Forum and Annual Congress of Chinese Society of Nephrology conferences from 2009 to 2014 were checked to identify additional studies. We also requested unpublished data and abstracts from pharmaceutical companies but no documents were provided.

#### 2.2.3. Data Selection, Data Extraction, and Quality Assessment

Two researchers (Yenan Mo and Xindong Qin) independently examined the articles according to the inclusion criteria and extracted data for collection. Disagreements were resolved by discussion and consensus and mediated by a third reviewer (Lixin Wang). The risk of bias was assessed using the Cochrane collaboration tool. The Grading of Recommendations Assessment, Development and Evaluation (GRADE) framework [41] was applied to assess the quality of evidence for each outcome, and the results were summarized in a Summary of Findings Table using GRADEpro 3.6 [42].

#### 2.2.4. Data Analysis

RevMan 5.2 [43] was used to analyze the results. Dichotomous and continuous data were presented as odds ratio (OR) and mean difference (MD), respectively, with 95% confidence interval (CI). Heterogeneity between trials was identified by the *χ* ^2^ test. When there was acceptable homogeneity (*P* > 0.1, *I* ^2^ ≤ 50%), the fixed-effect model was used for meta-analysis. When heterogeneity was significant (*P* ≤ 0.1, *I* ^2^ > 50%), it was analyzed with consideration of clinical factors, such as dose, time-point, and duration of follow-up, and methodological factors, such as randomization, allocation concealment, and blinding. Subgroup analysis was performed if heterogeneity was detected. A random-effects model was used to pool data in the absence of heterogeneity. Descriptive analysis was performed in cases of unacceptable heterogeneity. Sensitivity analysis was carried to evaluate the stability of results.

## 3. Results

### 3.1. Description of Studies

**Figure 1:**
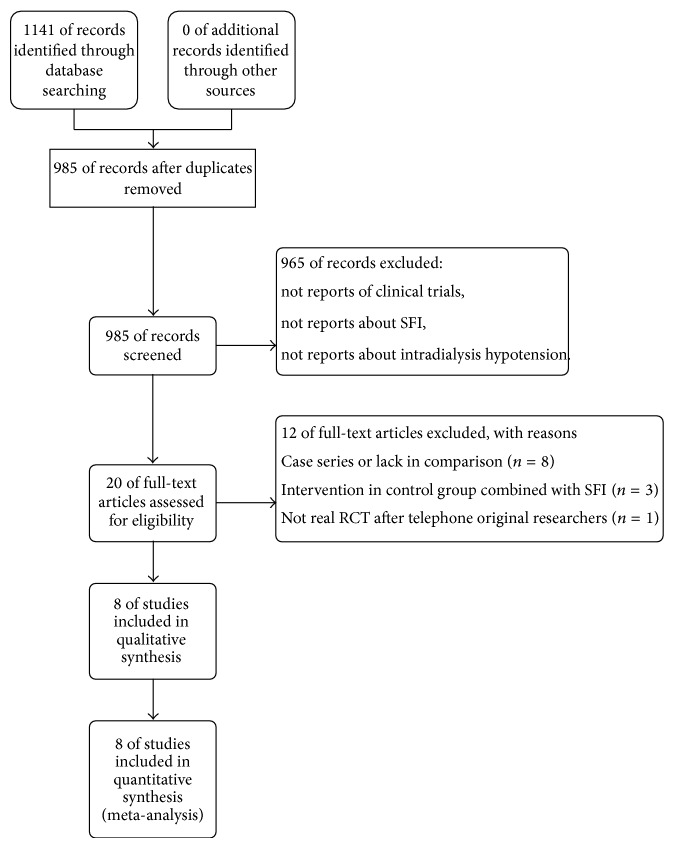


Of 1,141 potentially relevant articles identified and screened, 985 remained after duplicate records were deleted using Endnote X6. We excluded 965 articles based on the title and abstract because they were not reports of clinical trials or did not involve SFI or IDH. After reading the full text, eight articles were excluded as they were case reports or lacked a comparison group, two were excluded due to SFI existing in the control group, and one article was excluded because one of the authors revealed that it was not a valid RCT. Therefore, eight studies were included in the final analysis. Flow diagram was summarized in Figure 1.

### 3.2. Characteristics of the Included Studies

Of a total of 348 participants, 7,974 hemodialysis sessions were performed in the eight included studies (Table 1). All studies were conducted in China and published in Chinese between 2007 and 2013. The eight studies involved 200 males and 148 females with an age range of 21 to 83 years. The etiology of end-stage renal disease was identified in 248 patients in five studies, including 103 with chronic glomerulonephritis, 63 with diabetic nephropathy, 25 with obstructive nephropathy, 36 with hypertensive nephropathy, 8 with polycystic renal disease, 2 with chronic pyelonephritis, 3 with gouty nephropathy, 4 with lupus nephritis, and 4 with chronic interstitial nephritis. Participants in seven studies underwent bicarbonate dialysis for 4–4.5 h and 2-3 times per week. A flow-flux polysulfone hollow-fiber dialyzer was applied in six studies. Six studies reported the duration of dialysis as 2 months to 10 years. Of the interventions, conventional therapy referred to treatment according to K/DOQI guidelines [2], including changing dialysate temperature, adjusting sodium and ultrafiltration, increasing dialysate calcium, correcting anemia, and use of a vasoactive drug such as midodrine. The dose of SMI used ranged from 20 to 50 mL. SFI was administered intravenously in all included studies.

### 3.3. Risk of Bias in Included Studies (Figure 2)

All studies were of poor methodological quality and at high risk of bias. A random allocation was mentioned in all eight studies but none described the method used. The authors of three studies could not be contacted because of the wrong phone number [32], vacation [35], or dismissal [36]. After contacting the authors of the five remaining studies, it was determined that four studies [33, 34, 38, 39] used a random-number table and the remaining [37] study used computer software.

No study provided information about allocation concealment. After making phone calls to the authors, it was established that none of the studies adopted an adequate allocation concealment method.

Furthermore, blinding of key study personnel (patients, investigators, and assessors) was not used in any study.

Dropouts or withdrawals were not reported, and no trial reported an intention-to-treat analysis. All of the included studies appeared to have adequate and acceptable compliance.

The study protocols were not available, although the prespecified outcomes of four studies were reported in a prespecified manner [32, 33, 36, 37], while the other four studies utilized selective reporting [34, 35, 38, 39].

There may be a risk of other bias, but there was either insufficient information to assess whether an important risk of bias exists or insufficient evidence that an identified problem would introduce bias.

## 4. Synthesis of Results

### 4.1. Blood Pressure

**Figure 3:**
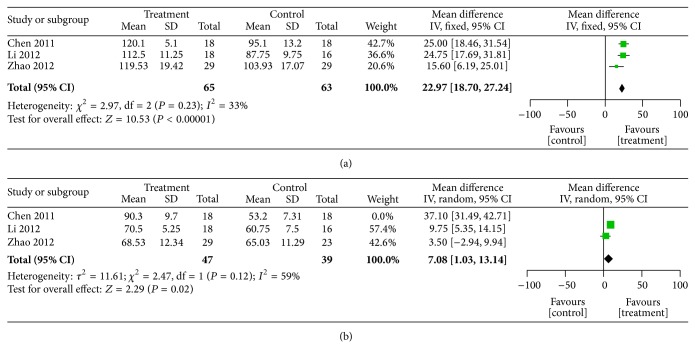
Forest plot of comparison: SFI versus control, (a) SBP, (b) DBP.

Three studies [32, 34, 38] reported pre- and post-SBP (Figure 3(a)) and diastolic blood pressure (Figure 3(b)). There was homogeneity in the SBP results (*χ* ^2^ = 2.97; *P* < 0.23; *I* ^2^ = 33%). Thus, a fixed-effects model was used for statistical analysis. There was a significant difference in SBP between the two groups (MD = 22.97; 95% CI, 18.70–27.24; *P* < 0.0001). However, the quality of Chen [32] was much lower than other two. So it was excluded for sensitive analysis. The heterogeneity was significant (*χ* ^2^ = 2.32; *P* = 0.13; *I* ^2^ = 57%). A random-effects model was used and the results were not changed materially (MD = 20.73; 95% CI, 11.82–29.63, *P* < 0.0001). So the SBP of meta-analysis was stable.

There was no homogeneity in the DBP results from (*χ* ^2^ = 76.46; *P* < 0.00001; *I* ^2^ = 97%). However, the heterogeneity could not be explained by clinical or methodological factors; thus, a random-effects model was used for statistical analysis. The increase in DBP compared with the control group was not statistically significant (MD = 16.80; 95% CI, −2.58–36.18; *P* < 0.09). The post-DBP in one study was considerably higher than those in two others; the demographic characteristics and the severity of disease could not account for the difference. Therefore, the study was excluded from the sensitivity analysis, which showed that heterogeneity remained (*χ* ^2^ = 2.47; *P* = 0.12; *I* ^2^ = 59%); the result was altered markedly (MD = 7.08; 95% CI, 1.03–13.14; *P* = 0.02). Therefore, the meta-analysis results were unstable.

### 4.2. Clinical Effective Rate

**Figure 4:**
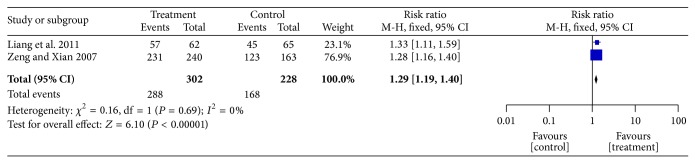
Forest plot of comparison: SFI versus control, the clinical effective rate.

Two trials [33, 37] calculated the clinical effective rate as the ratio between the proportion of responders in both groups (Figure 4). The two independent trials showed homogeneity in the trial results (*χ* ^2^ = 0.16; *P* = 0.69; *I* ^2^ = 0%). Thus, a fixed-effects model revealed that patients with IDH receiving SFI therapy had a significantly improved clinical effective rate compared with the control group (OR = 1.29; 95% CI, 1.19–1.40; *P* < 0.00001).

### 4.3. Incidence of Hypotension

**Figure 5:**
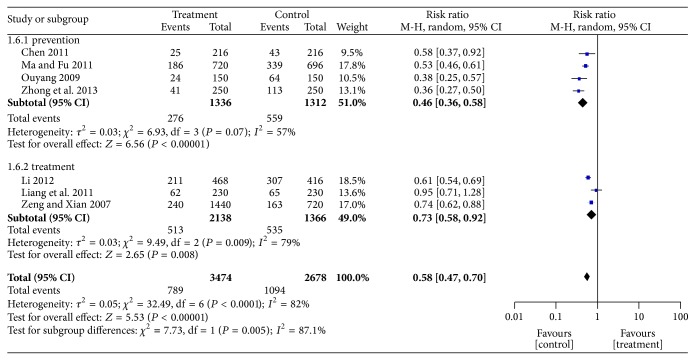
Forest plot of comparison: SFI versus control, the incidence of hypotension.

Seven studies evaluated the incidence of IDH episodes (Figure 5), and they did not show homogeneity (*χ* ^2^ = 30.33; *P* < 0.0001; *I* ^2^ = 80%). As SFI was used for prevention in four studies [32, 35, 36, 39] and treatment in another three [33, 34, 37], heterogeneity was explored by conducting a subgroup analysis. Heterogeneity was detected in both subgroups (*I* ^2^ = 57% in the prevention subgroup and *I* ^2^ = 77% in the treatment subgroup). Thus, a random-effects model was used for statistical analysis. SFI reduced the incidence of hypotension when used to prevent or treat an episode of IDH before or during dialysis (OR = 0.46; 95% CI, 0.36–0.58; *P* < 0.000001; OR = 0.72; 95% CI, 0.58–0.91; *P* < 0.005, resp.).

### 4.4. Serum Indices

**Figure 6:**
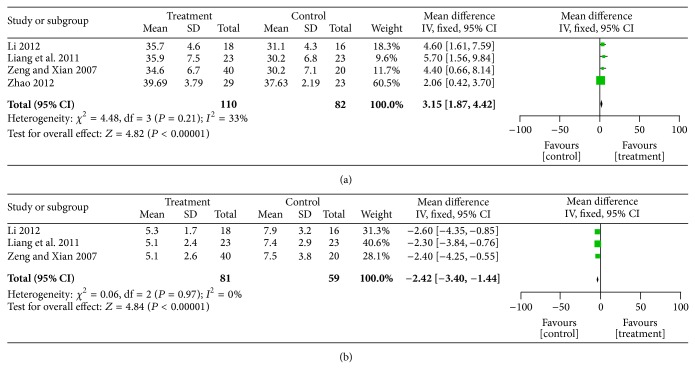
Forest plot of comparison: SFI versus control, (a) ALB; (b) CRP.

Four studies [33, 34, 37, 38] recorded ALB data before enrollment and at the completion of the study (Figure 6(a)). The heterogeneity was acceptable (*χ* ^2^ = 4.48; *P* = 0.21; *I* ^2^ = 33%), so a fixed-effects model was used for statistical analysis. SFI made a significant contribution to the elevated ALB in dialysis patients (MD = 2.06; 95% CI; 1.87,4.42; *P* < 0.00001). Three studies [33, 34, 37] recorded CRP data before the study began and after study completion (Figure 6(b)). The heterogeneity was excellent (*χ* ^2^ = 0.06; *P* = 0.97; *I* ^2^ = 0%), so a fixed-effects model was used for statistical analysis. SFI decreased the CRP level of dialysis patients (MD = −2.42; 95% CI; −3.40, −1.44; *P* < 0.00001). ALB is an important indicator of nutrient status, while CRP is predictive of cardiovascular events. Therefore, SFI may be used to increase the long-term survival rate of dialysis patients.

### 4.5. Adverse Events

An adverse event was reported in only one trial, which involved 50 patients. One patient complained of a headache and two had a rash that disappeared after 1 day [39]. Two trials reported no adverse effects in patients during observation [35, 37]. The other five trials did not report adverse effects.

### 4.6. Sensitivity Analysis

With the exception of DBP, each study was excluded from the sensitivity analysis, one by one. This did not materially change the results, which strengthened our confidence in their validity.

## 5. Discussion

### 5.1. Summary of the Main Results

Eight studies, involving a total of 348 individuals in 7974 HD sessions, were included in the present meta-analysis. The sample size varied from 30 to 60 participants. The main findings were that compared with conventional therapy alone, SFI adjuvant therapy resulted in an elevated DBP, increased clinical effectiveness rate, decreased incidence of hypotension, increased ALB, and decreased CRP without serious adverse effects. However, the evidence presented in the meta-analysis was insufficient to warrant a clinical recommendation due to the generally weak methodological quality of the included studies.

### 5.2. Overall Completeness and Applicability of Evidence

Our findings are generalizable to the majority of patients with IDH being treated with SFI in addition to conventional therapies, regardless of age, the cause of kidney function failure, and the duration of HD. SFI can prevent the occurrence of hypotension before dialysis and relieve the symptoms thereof during dialysis. However, the composition of Chinese herbal medicine is too complex to extract a single component, while the main ingredient of prepared aconite root is poisonous. Therefore, SFI cannot be accessed overseas, which may explain why all articles included in the meta-analysis were in the Chinese language and that SFI was used only in domestic hospitals.

### 5.3. Quality of Evidence

The Cochrane Collaboration Network GRADE was used to perform a systematic review of the results (Table 2). The systematic analysis contained seven outcomes. DBP and SBP were key outcomes, while the clinical effective rate, hypotension incidence, and ALB and CRP levels were important outcomes. The GRADE profile indicated that the quality of evidence of SBP, the effective rate, ALB, and CRP was low, and that of hypotension incidence and DBP was very low. Downgrading was due primarily to methodological limitations; the inconsistency could not be explained.

### 5.4. Limitations in the Review Process

#### 5.4.1. Methodological Quality

Firstly, the methodological quality of the studies was Poor. All studies claimed to be RCTs, but none described the randomization procedure. Furthermore, allocation concealment and blinding were not applied in any study. No studies reported dropout or loss-to-follow-up, and intention-to-treat was not mentioned. In addition, no protocol was available to confirm that studies were free of selective reporting bias. These different types of bias could therefore have led to false-positive results.

#### 5.4.2. Limited Outcomes

Secondly, limited outcomes were reported, especially with regard to adverse events and prognosis. Only 37.5% of the trials described the occurrence of adverse events, indicating an incomplete evaluation of the safety profile of SFI, as well as poor quality of reporting. In most trials, the duration of therapy was insufficient to yield conclusive results. Prepared aconite root is poisonous, but long-term toxicity was not monitored. Repeated episodes of IDH will influence the prognosis of HD patients, and some drugs increase mortality over the long-term despite a short-term improvement in clinical symptoms. However none of the studies involved a follow-up period after treatment. Therefore we were unable to adequately assess the effectiveness and safety of SFI for IDH.

#### 5.4.3. Inconsistent Interventions

Thirdly, the intervention should be compared with a placebo or the current “gold standard treatment.” However, IDH is an emergency that requires immediate intervention and there is no standard treatment other than conventional therapies. All studies included in this review used an “A+B versus B” design in which patients received conventional therapies alone or in combination with SFI. However, the type of conventional therapy varied among trials, which might have introduced bias.

#### 5.4.4. Publication Bias

The number of included articles did not allow the generation of a funnel plot to assess publication bias. In addition, all trials included in this paper were published in Chinese journals, which may limit the generalizability of the findings. All trials reported positive results, which are much easier to publish in China, and no study that reported a negative result was available, although we made an effort to include unpublished data. Therefore, publication bias might have existed.

#### 5.4.5. Lack of Economic Data

No economic data or a relative economic analysis has been reported.

## 6. Conclusions

### 6.1. Implications for Practice

This is the first meta-analysis of RCTs of the effectiveness and safety of SFI for patients with IDH. However, the evidence available from this systematic review is insufficient to recommend the routine use of SFI for IDH because of the methodological limitations of the included studies. Thus, the effectiveness and safety of SFI therapy for IDH remain to be determined.

### 6.2. Implications for Research

A sample size calculation should be conducted before enrollment. Randomization, allocation concealment, and blinding should be designed and carried out appropriately. In addition, the duration of follow-up should be sufficient to make adjustments for long-term effectiveness and safety, as well as to conduct a subgroup analysis. Furthermore, clinical trials should be registered in the WHO International Clinical Registry Platform in advance and reported in detail according to the CONSORT [44] or CONSORT for TCM [45] guidelines. More attention should be paid to side effects to comprehensively assess the safety of SFI. Finally, collection and reporting of relative economic data are necessary.

## Supplementary Material

Characteristics of all the mentioned studies, including methods, participants, interventions, outcomes, and notes, are tabulated for each study separately. Along with a review of authors' judgments about each risk of bias item presented as percentages across all included studies.

## Figures and Tables

**Figure 2:**
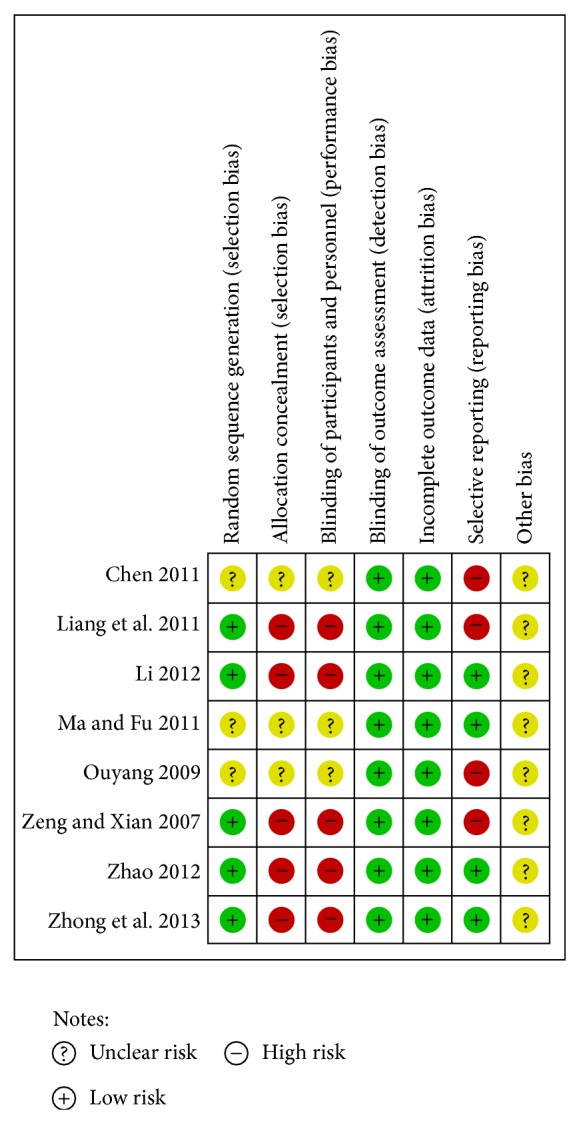
Risk of bias summary.

**Table 1:** Characteristics of included studies.

Studies	Methods	Participants			Cause of Renal Failure	Intervention		Outcomes	Follow-up
		*N* (T/C)	Age	M/F		T	C		
Chen 2011 [32]	D: RCT R: NM A: NM B: NM	36 (18/18, 216/216^*^)	(43.2 ± 10.2) (42.6 ± 11.4)	11/17	NM	00:00 SFI 20 mL + 0.9% NS 130 mL	00:00 0.9% NS 150 mL	HI, BP, Dry weight	4 w

Liang et al., 2011 [33]	D: RCT R: Random-number table A: N B: N	46 (23/23, 240/240^*^)	(49.5 ± 7.6) (48.2 ± 7.2)	31/15	21 CGN 13 DN, 7 ON, 3 HN, 2 GN	SOS: SFI 40 mL + CT	SOS: CT	BP, HR, ALB, CRP, Clinical effect	3-4 w

Li 2012 [34]	D: RCT R: Random- number table A: N B: N	34 (18/16, 468/416^*^)	(54.7 ± 13.5) (56.3 ± 12.7)	23/11	NM	SOS: SFI 40 mL + CT	SOS: CT	HI, SBP, DBP, ALB, CRP.	12 w

Ma and Fu 2011 [35]	D: RCT R: NM A: NM B: NM	40 (20/20, 720/696^*^)	29–72 (56.6) 30–74 (56.8)	27/13	20 CGN 6 DN, 3 ON, 9 HN, 2 O	00:00 SFI 40 mL SOS: CT	SOS: CT	HI, Clinical symptom	12 w

Ouyang 2009 [36]	D: RCT R: NM A: NM B: NM	30 (15/15, 150/150^*^)	(52.3 ± 17.2) (53.6 ± 17.9)	13/17	NM	02:00 SFI 50 mL + 0.9% NS 250 mL	02:00 0.9% NS 300 mL	BP, CS, Intervention for IDH	3–5 w

Zeng and Xian 2007 [37]	D: RCT R: Computer Software A: N Bi: N	60 (40/20, 1440/720^*^)	(46.8 ± 13.7) (44.1 ± 12.3)	33/27	28 CGN 11 DN, 12 ON, 3 HN, 6 O	SOS: SFI 20 mL + 50 g/L GS 30 mL	SOS: 50 g/L GS 30 mL	BP, HR, ALB, SE, HI, CRP, Serum osmolality	12 w

Zhao 2012 [38]	D: RCT R: Random- number table A: N B: N	52 (29/23)	(69.48 ± 6.43) (69.65 ± 6.73)	33/19	27 CGN 9 DN, 3 ON, 7 HN, 6 O	00:00 SFI 50 mL + 0.9% NS 50 mL	00:00 0.9% NS 100 mL	BP, ALB, SCR, HI, BUN, HB, ultrafiltration volume	12 w

Zhong et al., 2013 [39]	D: RCT R: Random- number table A: N B: N	55 (25/25, 250/250^*^)	(65.3 ± 14.2) (62.6 ± 16.7)	23/27	7 CGN, 24 DN, 14 HN, 1 GN, 6 O	00:10 & 02:00: SFI 20 mL + 50% GS 20 mL	00:10 & 02:00 0.9% NS 40 mL	CS, Side effect.	3–5 w

Notes: D: design; R: randomization; A: allocation concealment; B: binding; T/C: treatment group/control group; M/F: male/female; CGN: chronic glomerulonephritis; DN: diabetic nephropathy; ON: obstructive nephropathy; HN: hypertension nephropathy; O: others. HR: heart rate; HI: hypotension incidence; CS: clinical symptom; ^*^hemodialysis sessions.

SOS: when hypotension happened during dialysis. 00:00, 00:10, 02:00: the time recorded as dialysis started.

**Table 2:** Grade Quality of evidence of SFI for IDH.

Outcomes	Illustrative comparative risks^*^ (95% CI)		Relative effect (95% CI)	No of Participants (studies)	Quality of the evidence (GRADE)
	Assumed risk	Corresponding risk			
	Control	Shenfu			
**Systolic blood pressure** Follow-up: 3–12 w		The mean systolic blood pressure in the intervention groups was **22.64 higher** (17.35 to 27.94 higher)		128 (3 studies)	*⊕⊕* *⊝⊝* **Low** ^1^

**Diastolic blood pressure** Follow-up: 3–12 w		The mean diastolic blood pressure in the intervention groups was **16.8 higher** (2.59 lower to 36.18 higher)		122 (3 studies)	*⊕* *⊝⊝* *⊝* **Very low** ^1,2^

**The clinical effective rate** Follow-up: 3–12 w	**Study population**		**RR 1.29** (1.19 to 1.4)	530 (2 studies)	*⊕⊕* *⊝⊝* **Low** ^1^
	**737 per 1000**	**951 per 1000** (877 to 1000)			
	**Moderate**				
	**724 per 1000**	**934 per 1000** (862 to 1000)			

**Hypotension incidence**	**Study population**		**RR 0.57** (0.47 to 0.7)	7348 (7 studies)	
	**334 per 1000**	**190 per 1000** (157 to 234)			
	**Moderate**				
	**427 per 1000**	**243 per 1000** (201 to 299)			

**Hypotension incidence-Prevention** Follow-up: 3–12 w	**Study population**		**RR 0.46** (0.36 to 0.58)	2648 (4 studies)	*⊕* *⊝⊝* *⊝* **Very low** ^1,2^
	**426 per 1000**	**196 per 1000** (153 to 247)			
	**Moderate**				
	**439 per 1000**	**202 per 1000** (158 to 255)			

**Hypotension incidence-Treatment** Follow-up: 3–12 w	**Study population**		**RR 0.72** (0.58 to 0.91)	4700 (3 studies)	*⊕* *⊝⊝* *⊝* **Very low** ^1,2^
	**272 per 1000**	**196 per 1000** (158 to 248)			
	**Moderate**				
	**226 per 1000**	**163 per 1000** (131 to 206)			

**ALB** Follow-up: 3 months		The mean alb in the intervention groups was **3.15 higher** (1.87 to 4.42 higher)		192 (4 studies)	*⊕⊕* *⊝⊝* **Low** ^1^

**CRP** Follow-up: 3 months		The mean crp in the intervention groups was **2.42 lower** (3.4 to 1.44 lower)		140 (3 studies)	*⊕⊕* *⊝⊝* **Low** ^1^

^1^None of the trials were blinded or allocation concealed.

^2^Confidence intervals with minimal overlap, the *P* value for heterogeneity is less than 0.05 and *I* ^2^ > 50%. Heterogeneity was not explained.

^*^Means hemodialysis sessions, as they need dialysis twice or thrice in every week.

## References

[1] Palmer B. F., Henrich W. L. (2008). Recent advances in the prevention and management of intradialytic hypotension. *Journal of the American Society of Nephrology*.

[2] K/DOQI Workshop (2005). K/DOQI clinical practice guidelines for cardiovascular disease in dialysis patients. *The American Journal of Kidney Diseases*.

[3] Hayes W., Hothi D. K. (2011). Intradialytic hypotension. *Pediatric Nephrology*.

[4] Toyoda K., Fujii K., Fujimi S., Kumai Y., Tsuchimochi H., Ibayashi S., Iida M. (2005). Stroke in patients on maintenance hemodialysis: a 22-year single-center study. *American Journal of Kidney Diseases*.

[5] John A. S., Tuerff S. D., Kerstein M. D. (2000). Nonocclusive mesenteric infarction in hemodialysis patients. *Journal of the American College of Surgeons*.

[6] Mcintyre C. W. (2010). Recurrent circulatory stress: the dark side of dialysis. *Seminars in Dialysis*.

[7] Wizemann V., Wabel P., Chamney P. (2009). The mortality risk of over-hydration in haemodialysis patients. *Nephrology Dialysis Transplantation*.

[8] Jansen M. A. M., Hart A. A. M., Korevaar J. C., Dekker F. W., Boeschoten E. W., Krediet R. T. (2002). Predictors of the rate of decline of residual renal function in incident dialysis patients. *Kidney International*.

[9] Chang T. I., Paik J., Greene T., Desai M., Bech F., Cheung A. K., Chertow G. M. (2011). Intradialytic hypotension and vascular access thrombosis. *Journal of the American Society of Nephrology*.

[10] Agarwal R. (2012). How can we prevent intradialytic hypotension?. *Current Opinion in Nephrology and Hypertension*.

[11] Zhou Y. L., Liu H. L., Duan X. F., Yao Y., Sun Y., Liu Q. (2006). Impact of sodium and ultrafiltration profiling on haemodialysis-related hypotension. *Nephrology Dialysis Transplantation*.

[12] Genovesi S., Dossi C., Viganò M. R., Galbiati E., Prolo F., Stella A., Stramba-Badiale M. (2008). Electrolyte concentration during haemodialysis and QT interval prolongation in uraemic patients. *Europace*.

[13] Damasiewicz M. J., Polkinghorne K. R. (2011). Intra-dialytic hypotension and blood volume and blood temperature monitoring. *Nephrology*.

[14] Nesrallah G. E., Suri R. S., Guyatt G., Mustafa R. A., Walter S. D., Lindsay R. M., Akl E. A. (2013). Biofeedback dialysis for hypotension and hypervolemia: a systematic review and meta-analysis. *Nephrology Dialysis Transplantation*.

[15] Prakash S., Garg A. X., Heidenheim A. P., House A. A. (2004). Midodrine appears to be safe and effective for dialysis-induced hypotension: a systematic review. *Nephrology Dialysis Transplantation*.

[16] Lynch K. E., Feldman H. I., Berlin J. A., Flory J., Rowan C. G., Brunelli S. M. (2008). Effects of l-carnitine on dialysis-related hypotension and muscle cramps: a meta-analysis. *The American Journal of Kidney Diseases*.

[17] Locatelli F., Altieri P., Andrulli S., Bolasco P., Sau G., Pedrini L. A., Basile C., David S., Feriani M., Montagna G., Di Iorio B. R., Memoli B., Cravero R., Battaglia G., Zoccali C. (2010). Hemofiltration and hemodiafiltration reduce intradialytic hypotension in ESRD. *Journal of the American Society of Nephrology*.

[18] Weinreich T., de los Ríos T., Gauly A., Passlick-Deetjen J. (2006). Effects of an increase in time vs. frequency on cardiovascular parameters in chronic hemodialysis patients. *Clinical Nephrology*.

[19] Jefferies H. J., Virk B., Schiller B., Moran J., Mcintyre C. W. (2011). Frequent hemodialysis schedules are associated with reduced levels of dialysis-induced cardiac injury (myocardial stunning). *Clinical Journal of the American Society of Nephrology*.

[20] Zhao X. K. (2009). *Thorough Knowledge of Medicine*.

[21] Yan Y. H. (1980). *Rearrangement Edition of Yan's Prescriptions for Rescuing Lives*.

[22] Xu J., Lou H. G., Lou Y. J. (2008). Research advance in pharmacological action of shenfu injection. *Shanghai Journal of Traditional Chinese Medicine*.

[23] Shen Y. J. (2000). *Traditional Chinese Medicine Pharmacology*.

[24] Ji X.-F., Yang L., Zhang M.-Y., Li C.-S., Wang S., Cong L.-H. (2011). Shen-Fu injection attenuates postresuscitation myocardial dysfunction in a porcine model of cardiac arrest. *Shock*.

[25] Xia S. Y., Zheng L. W. (2011). Experiment study of Shenfu Injecion protecting Intestinal mucosa from ischemic re-perfusion injury. *Chinese Journal of Traumatology*.

[26] Liu Z., Xie G. Q. (2011). The Therapeutic Tactics on Hemodialysis-induced Hypotension. *Journal of New Chinese Medicine*.

[27] Li Q. B., Liu X. (1999). Shenfu Injection in the clinical application of the rescue of severe hemorrhagic shock. *Journal of Guangdong Medical College*.

[28] Hu J., Fu Z.-Y., Xie Y.-M., Wang J., Wang W.-W., Liao X. (2013). Systematic review of Shenfu injection for septic shock. *Zhongguo Zhongyao Zazhi*.

[29] Tian J.-F., Li J.-D., Lei Y. (2012). Clinical features of acute myocardial infarction inpatients in 26 level three class A Chinese medicine hospitals in China and the investigation of treatment. *Zhongguo Zhong Xi Yi Jie He Za Zhi*.

[30] Liu J. W., Dai Y. P. (2010). Introduction of pharmacological action of Shenfu injection and clinical practice in cardiovascular disease. *Journal of Liaoning University of Traditional Chinese Medicine*.

[31] Hou Y. Z., Mao J. Y., Wang X. L., Li J., Liu C. X. (2011). Shenfu injection for patients with heart failure: a systematic review. *Chinese Journal of Evidence-Based Medicine*.

[32] Chen Y. Q. (2011). The application of Shenfu injection pre-rinsing pipeline hemodialysis-related hypotension. *Chinese Journal of Chinese Medicine*.

[33] Liang R. J., Lu Y. M.,  Li X. H. (2011). The clinical observation of midodrine united with shenfu injection treating with hemodialysis—related hypopiesia. *China Medical Engineering*.

[34] Li Z. H. (2012). Effects of Shenfu injection and compound amino acid injection on hypotension of uremia cases undergoing hemodialysis. *Journal of Qiqihar University of Medicine*.

[35] Ma L. L., Fu L. Y. (2011). Clinical observation of Shenfu Injection in preventing and treating dialysis induced syncope. *Journal of Emergency in Traditional Chinese Medicine*.

[36] Ouyang B. (2009). Clinical observation of Shenfu Injection in preventing and treating hemodialysis hypotension. *Chinese Journal of Nephrology of Integrated*.

[37] Zeng L., Xian Q. J. (2007). Shenfu Injection for the treatment of hemodialysis induced hypotension. *Guangdong Medical Journal*.

[38] Zhao X. F. (2012). Clinical observation of Shenfu Injection in preventing and treating hemodialysis hypotension. *Chinese Journal of Information on TCM*.

[39] Zhong J., Xiong W. J., Yang J. (2013). Observation of the curative effect of Shenfu injection on hemodialysis associated hypotension of the elderly. *Journal of Emergency in Traditional Chinese Medicine*.

[40] Wang Z. G. (2010). *Blood Purification*.

[41] GRADE Working Group (2004). Grading quality of evidence and strength of recommendations. *British Medical Journal*.

[42] Schünemann H., Brożek J., Oxman A. (2009). *GRADE Handbook for Grading Quality of Evidence and Strength of Recommendation*.

[43] RevMan (2012). *[Computer program]. Version 5.2.*.

[44] Schulz K. F., Altman D. G., Moher D. (2010). CONSORT 2010 statement: updated guidelines for reporting parallel group randomised trials. *PLoS Medicine*.

[45] Wu T.-X., Li Y.-P., Bian Z.-X., Li T.-Q., Li J., Dagenais S., Moher D. (2007). Consolidated standards for reporting trials of traditional Chinese medicine (CONSORT for TCM) (for solicitation of comments). *Chinese Journal of Evidence-Based Medicine*.

