# Supplementary material for: Shenfu Injection for Intradialytic Hypotension: A Systematic Review and Meta-Analysis
Source: Evid Based Complement Alternat Med. 2014 Dec 22;2014:279853. doi: 10.1155/2014/279853 (PMC4284991; doi:10.1155/2014/279853)
Supplement: Supplementary file 1 — Characteristics of all the mentioned studies, including methods, participants, interventions, outcomes, and notes, are tabulated for each study separately. Along with a review of authors' judgments about each risk of bias item presented as percentages across all included studies. [file 279853.f1.docx]

## Characteristics of included studies

### Chen YQ 2011

| Methods | Design: RCT  Randomization:not stated.  Allocation concealment: not stated.  Blinding:not stated. |
| --- | --- |
| Participants | Site: Second People Hospital of Qujing City, Yunnan  N=36 (T/C:18/18, 216/216* )  Age: T: (43.2±10.2) C: (42.6±11.4)  Sex: M/F=11/17  Time of dialysis: 5 months - 6 years, 4hrs, 3/week  Cause of renal failure: not stated  Dialysis equipment: Dialog, F6 Polysulfone, Bicarbonate |
| Interventions | Experiment group: SFI 20mL+ 0.9% sodium chloride solution 130mL prerinsing pipeline  Control group: 0.9% sodium chloride solution prerinsing pipeline |
| Outcomes | Hypotension incidence, SBP, DBP, Dry weight. |
| Notes | Manufacturer of SFI：not stated. |

#### Risk of bias table

| **Bias** | **Authors' judgement** | **Support for judgement** |
| --- | --- | --- |
| Random sequence generation (selection bias) |  | 'Randomised' - no further description,and the author couldn't be contacted because of the wrong number. |
| Allocation concealment (selection bias) |  | Not described,and the author couldn't be contacted because of the wrong number. |
| Blinding of participants and personnel (performance bias) |  | Not described. |
| Blinding of outcome assessment (detection bias) |  | Not described. As a primary outcome, BP was a objective outcome measured by hemopiezometer. |
| Incomplete outcome data (attrition bias) |  | No drop-up data were mentioned, while dialysis patients generally had adequate and acceptable compliance. |
| Selective reporting (reporting bias) |  | Data about symptom were not reported. |
| Other bias |  | Funding source: not stated. |

### Li ZH 2012

| **Methods** | Design: RCT  Randomization:random-number table.  Allocation concealment: no.  Blinding:no |
| --- | --- |
| **Participants** | Site: Central Hospital of Longgang, Shenzhen  N=34 (T/C:18/16, 468/416* )  Age:T: (54.7±13.5) C: (56.3±12.7)  Sex: M/F=23/11  Time of dialysis: 1- 5 years, 4hrs, 2/week.  Cause of renal failure: not stated  Dialysis equipment: F6 Polysulfone, Bicarbonate. |
| **Interventions** | Experiment group: Conventional therapy + SFI 40mL iv  Control group: Conventional therapy (dorsal elevated position, slow down blood flow, oxygen inhalation therapy). |
| **Outcomes** | Hypotension incidence,SBP,DBP,ALB,CRP. |
| **Notes** | Manufacturer of SFI：not stated. |

#### Risk of bias table

| **Bias** | **Authors' judgement** | **Support for judgement** |
| --- | --- | --- |
| Random sequence generation (selection bias) |  | 'Randomised' - random-number table. |
| Allocation concealment (selection bias) |  | No |
| Blinding of participants and personnel (performance bias) |  | No. |
| Blinding of outcome assessment (detection bias) |  | No. As a primary outcome, BP was a objective outcome measured by hemopiezometer. |
| Incomplete outcome data (attrition bias) |  | No drop-up data were mentioned, while dialysis patients generally had adequate and acceptable compliance. |
| Selective reporting (reporting bias) |  | All outcomes reported. |
| Other bias |  | Funding source: not stated. |

### Liang RJ 2011

| **Methods** | Design: RCT  Randomization:random-number table.  Allocation concealment: no.  Blinding:no |
| --- | --- |
| **Participants** | Site: People Hospital of Maoming City, Guangdong  N=46 (T/C:23/23,240/240* )  Age: T: (49.5±7.6) C: (48.2±7.2)  Sex: M/F=31/15  Cause of renal failure: 21 CGN, 13 DN, 7 ON, 3 HTN, 2 GN  Time of dialysis: not stated duration , 4hrs, 3/week.  Dialysis equipment: F60, F6 Polysulfone, Bicarbonate. |
| **Interventions** | Experimental group:30min before dialysis: Midodrine hydrochloride Tablet 5mg po. When hypotension happened: conventional therapy + SFI 40mL iv.  Control group:30min before dialysis: Midodrine hydrochloride Tablet 5mg po. When hypotension happened: conventional therapy (reduce ultrafiltration rate, increase sodium concentration in dialyzate, change dialyzate temperature, infuse NS or hypertonic GS) |
| **Outcomes** | SBP,DBP,HR,Clinical effect,ALB,CRP. |
| **Notes** | Manufacturer of SFI：not stated. |

#### Risk of bias table

| **Bias** | **Authors' judgement** | **Support for judgement** |
| --- | --- | --- |
| Random sequence generation (selection bias) |  | 'Randomised' -random-number table. |
| Allocation concealment (selection bias) |  | No |
| Blinding of participants and personnel  (performance bias) |  | No |
| Blinding of outcome assessment  (detection bias) |  | No. As a primary outcome, BP was a objective outcome measured by hemopiezometer. |
| Incomplete outcome data (attrition bias) |  | No drop-up data were mentioned, while dialysis patients generally had adequate and acceptable compliance. |
| Selective reporting (reporting bias) |  | The data about heart rate were not reported. |
| Other bias |  | Funding source: not stated. |

### Ma LL2011

| **Methods** | Design: RCT  Randomization:not stated.  Allocation concealment:not stated.  Blinding:not stated. |
| --- | --- |
| **Participants** | Site: Shaoxing Hospital of TCM, Zhejiang  N=40 (T/C:20/20, 720/696* )  Age: T: 29-72(56.6) C: 30-74(56.8)  Sex:M/F=27/13  Time of dialysis: not stated duration, 4.5hrs, 2/week.  Cause of renal failure: 20 CGN, 6 DN, 3 ON, 9 HTN, 2 PRD  Dialysis equipment: Polysulfone, Bicarbonate |
| **Interventions** | Experiment group: When dialysis beginning and maintain for 1-2h: SFI 40mL iv .When IDH happened: conventional therapy.  Control group:When IDH happened: conventional therapy (reduce ultrafiltration rate, slow down blood flow) |
| **Outcomes** | Clinical symptom, Hypotension incidence. |
| **Notes** | Manufacturer of SFI: Yaan Sanjiu Medicine |

| **Bias** | **Authors' judgement** | **Support for judgement** |
| --- | --- | --- |
| Random sequence generation (selection bias) |  | 'Randomised' - no further description. |
| Allocation concealment (selection bias) |  | Not described. |
| Blinding of participants and personnel (performance bias) |  | Not described. |
| Blinding of outcome assessment (detection bias) |  | Not described.As a primary outcome, BP was a objective outcome measured by hemopiezometer.. |
| Incomplete outcome data (attrition bias) |  | No drop-up data were mentioned, while dialysis patients generally had adequate and acceptable compliance. |
| Selective reporting (reporting bias) |  | All outcomes reported. |
| Other bias |  | Funding source: not stated. |

#### Risk of bias table

### OY B 2009

| **Methods** | Design: RCT  Randomization:not stated.  Allocation concealment:not stated.  Blinding:not stated. |
| --- | --- |
| **Participants** | Site: The first affiliated hospital of Tianjin University of TCM  N=30 (T/C:15/15, 150/150* )  Age: T: (52.3±17.2) C: (53.6±17.9)  Sex: M/F=13/17  Time of dialysis: .3 months - 8 years, 4hrs, 2-3/week.  Cause of renal failure: not stated  Dialysis equipment:not stated. |
| **Interventions** | Experiment group:SFI 50ml+0.9% NS 250mL ivd 40gtt/min at the second hour of dialysis  Control group:When IDH happened: conventional therapy (reduce ultrafiltration rate, slow down blood flow) |
| **Outcomes** | SBP,DBP,clinical symptom,the interventions for IDH |
| **Notes** | Manufacturer of SFI: Wangrong Sanjiu Medicine |

#### Risk of bias table

| **Bias** | **Authors' judgement** | **Support for judgement** |
| --- | --- | --- |
| Random sequence generation (selection bias) |  | 'Randomised' - no further description. |
| Allocation concealment (selection bias) |  | Not described. |
| Blinding of participants and personnel (performance bias) |  | Not described. |
| Blinding of outcome assessment (detection bias) |  | As a primary outcome, BP was a objective outcome measured by hemopiezometer. |
| Incomplete outcome data (attrition bias) |  | No drop-up data were mentioned, while dialysis patients generally had adequate and acceptable compliance. |
| Selective reporting (reporting bias) |  | Data about symptom and interventions when hypotension happened . |
| Other bias |  | Funding source: not stated. |

### Zeng L 2007

| **Methods** | Design: RCT  Randomization:Computer software.  Allocation concealment: no.  Blinding: no | |
| --- | --- | --- |
| **Participants** | | Site: The first affiliated hospital of Guangzhou University of TCM  N=60 (T/C:40/20, 1440/720*)  Age: T: (46.8±13.7) C: (44.1±112.3)  Sex: M/F=33/27  Time of dialysis: 13 - 60 months, 4hrs, 3/week.  Cause of renal failure: 28 CGN,11 DN, 12 ON, 3 HTN, 2 PRD, 2 LN, 2 CIN.  Dialysis equipment: Polysulfone, Bicarbonate. |
| **Interventions** | | Experiment group: When IDH happened,50g/L GS 30mL (3% GS 30mL for diabetes patients) + SFI 20mL iv.  Control group: When IDH happened.50g/L GS 30mL (3% GS 30mL for diabetes patients) iv. |
| **Outcomes** | | SBP, DBP,HR,ALB,Hypotension incidence,Serum osmolality,CRP,Side effects |
| **Notes** | | Manufacturer of SFI: Guangdong Sanjiu Medicine |

| **Bias** | **Authors' judgement** | **Support for judgement** |
| --- | --- | --- |
| Random sequence generation (selection bias) |  | 'Randomised' - Computer software. |
| Allocation concealment (selection bias) |  | No |
| Blinding of participants and personnel (performance bias) |  | No |
| Blinding of outcome assessment (detection bias) |  | As a primary outcome, BP was a objective outcome measured by hemopiezometer. |
| Incomplete outcome data (attrition bias) |  | No drop-up data were mentioned, while dialysis patients generally had adequate and acceptable compliance. |
| Selective reporting (reporting bias) |  | The data about serum osmolality were not reported. |
| Other bias |  | Funding source: not stated. |

#### Risk of bias table

### Zhao XF 2012

| **Methods** | Design: RCT  Randomization:random-number table.  Allocation concealment: no.  Blinding: no |
| --- | --- |
| **Participants** | Site: Liuzhou Hospital of TCM  N=52 (T/C:29/23 )  Age:T: (69.48±6.43) C: (69.65±6.73)  Sex: M/F=33/19  Time of dialysis: .16 - 68 months, 4hrs, 3/week.  Cause of renal failure: 27 CGN, 9 DN, 3 ON, 7 HN, 2 PRD, 2 LN, 2 CIN.  Dialysis equipment: Fresenius Polysulfone, Bicarbonate. |
| **Interventions** | Experiment group: Along with dialysis, SFI 50mL+ 0.9% NS 50mL ivd  Control group: Along with dialysis, 0.9% NS 100 mL ivd . |
| **Outcomes** | BP, ultrafiltration volume, Hypotension incidence, the number of continuing or discontinuing dialysis after intention,ALB, SCR, BUN,Na+,HB |
| **Notes** | Manufacturer of SFI: Yaan Sanjiu Medicine |

#### Risk of bias table

| **Bias** | **Authors' judgement** | **Support for judgement** |
| --- | --- | --- |
| Random sequence generation (selection bias) |  | 'Randomised' - random-number table. |
| Allocation concealment (selection bias) |  | No. |
| Blinding of participants and personnel (performance bias) |  | No. |
| Blinding of outcome assessment (detection bias) |  | No. As a primary outcome, BP was a objective outcome measured by hemopiezometer. |
| Incomplete outcome data (attrition bias) |  | No drop-up data were mentioned, while dialysis patients generally had adequate and acceptable compliance. |
| Selective reporting (reporting bias) |  | All outcomes reported. |
| Other bias |  | Funding source: not stated. |

### Zhong J 2013

| **Methods** | | Design: RCT  Randomization:random-number table.  Allocation concealment: no.  Blinding: no |
| --- | --- | --- |
| **Participants** | Site: Chongqing Hospital of TCM  N=550(T/C:25/25, 250/250* )  Age:T: (65.3±14.2) C: (62.6±16.7)  Sex: M/F=23/27  Time of dialysis: 2 months - 10 years, 4hrs, 3/week.  Cause of renal failure: 7 CGN, 24 DN, 1GN,14 HN, 2 PRD, 2 CPN, 2CPN.  Dialysis equipment: Bicarbonate | |
| **Interventions** | | Experiment group: At the time of 10 min and 2h of dialysis, SFI 20mL +50% GS (0.9% GS for diabetes patients) 20mL iv.  Control group:At the time of 10 min and 2h of dialysis.0.9% NS 40mL iv. |
| **Outcomes** | | Clinical symptom,Side effect. |
| **Notes** | | Manufacturer of SFI: Sanjiu Medicine |

#### Risk of bias table

| **Bias** | **Authors' judgement** | **Support for judgement** |
| --- | --- | --- |
| Random sequence generation (selection bias) |  | 'Randomised' - random-number table. |
| Allocation concealment (selection bias) |  | No. |
| Blinding of participants and personnel (performance bias) |  | No. |
| Blinding of outcome assessment (detection bias) |  | As a primary outcome, BP was a objective outcome measured by hemopiezometer. |
| Incomplete outcome data (attrition bias) |  | No drop-up data were mentioned, while dialysis patients generally had adequate and acceptable compliance. |
| Selective reporting (reporting bias) |  | All outcomes reported. |
| Other bias |  | Funding source: not stated. |


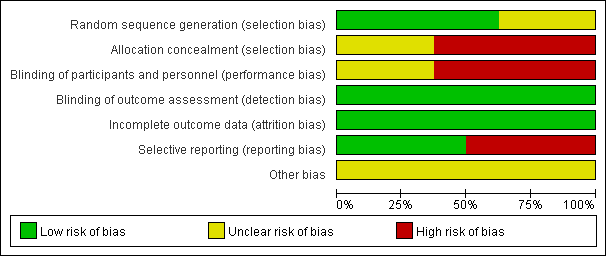
Risk of bias graph: review authors' judgements about each risk of bias item presented as percentages across all included studies.


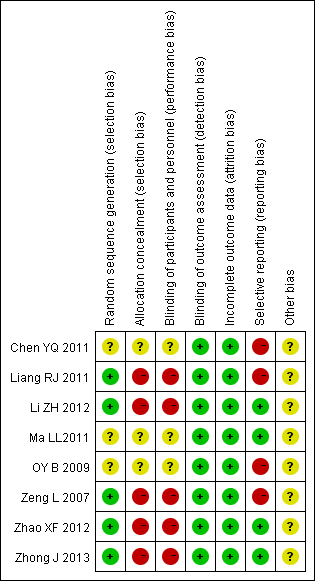


Risk of bias summary: review authors' judgements about each risk of bias item for each included study.
